# Clinical outcomes of adjuvant nivolumab in resected stage III melanoma: comparison of CheckMate 238 trial and real-world data

**DOI:** 10.1007/s00262-024-03697-3

**Published:** 2024-05-07

**Authors:** Justin C. Moser, Shailender Bhatia, Asim Amin, Anna C. Pavlick, Keith A. Betts, Ella Xiaoyan Du, Tayla Poretta, Karishma Shelley, Swetha Srinivasan, Leon Alan Sakkal, Jennell Palaia, Maurice Lobo, Melanie Pe Benito, Joshua A. Linton, Yan Chen, Churong Xu, Lei Yin, Manasvi Sundar, Jeffrey Weber

**Affiliations:** 1 HonorHealth Research Institute, 10510 North 92nd Street, Suite 100, Scottsdale, AZ 85258 USA; 2 Division of Hematology and Oncology, Fred Hutchinson Cancer Center, Seattle, WA USA; 3 Medical Oncology and Immunotherapy, Levine Cancer Institute, Atrium Health, Charlotte, NC USA; 4 Medical Oncology, Weill Cornell Medicine, New York, NY USA; 5 Analysis Group, Los Angeles, CA USA; 6 Bristol Myers Squibb, Princeton, NJ USA; 7 NYU Langone Health Perlmutter Cancer Center, New York, NY USA

**Keywords:** Adjuvant therapy, Immunotherapy, Nivolumab, Melanoma, Real-world

## Abstract

**Objectives:**

Nivolumab is approved as adjuvant therapy for resected stage III/IV melanoma based on the phase 3 CheckMate 238 trial. This analysis compared outcomes from CheckMate 238 with those from the real-world Flatiron Health electronic health record-derived de-identified database in patients with resected stage III melanoma (per AJCC-8) treated with adjuvant nivolumab.

**Materials:**

Outcomes included baseline characteristics, overall survival (OS) in the CheckMate 238 cohort (randomization until death or last known alive), and real-world overall survival (rwOS) in the Flatiron Health cohort (nivolumab initiation until death or data cutoff). rwOS was compared with OS using unadjusted and adjusted Cox proportional hazards models. Inverse probability of treatment weighting (IPTW) was combined with the adjusted model to reduce baseline discrepancies.

**Results:**

The CheckMate 238 and real-world cohorts included 369 and 452 patients, respectively (median age, 56.0 and 63.0 years; median follow-up, 61.4 vs. 25.5 months). rwOS was not different from OS in the unadjusted (hazard ratio [HR] 1.27; 95% CI 0.92–1.74), adjusted (HR 1.01; 95% CI 0.67–1.54), and adjusted IPTW (HR 1.07; 95% CI 0.70–1.63) analyses. In the adjusted analysis, 2-year OS and rwOS rates were 84%. Median OS and rwOS were not reached. After IPTW, OS and rwOS were not different (HR 1.07; 95% CI 0.70–1.64).

**Conclusions:**

In this comparative analysis, OS in the CheckMate 238 trial was similar to rwOS in the Flatiron Health database after adjustments in patients with resected stage III melanoma (per AJCC-8) treated with adjuvant nivolumab, validating the trial results.

**Supplementary Information:**

The online version contains supplementary material available at 10.1007/s00262-024-03697-3.

## Introduction

Systemic therapies indicated for patients with completely resected stage III or IV melanoma in the adjuvant setting include the immuno-oncology (I-O) agents nivolumab and pembrolizumab, as well as the BRAF plus MEK inhibitor combination of dabrafenib plus trametinib (for *BRAF*-mutant disease) [1]. Nivolumab, an anti-programmed cell death-1 (PD-1) antibody, is approved in the United States and other countries as adjuvant therapy for resected stage III or IV melanoma based on evidence from the phase 3 CheckMate 238 randomized controlled trial (RCT), which included patients with in-transit metastasis with and without nodal involvement [2]. In that trial, patients with stage IIIB, stage IIIC, or stage IV resected melanoma (per American Joint Committee on Cancer, *Cancer Staging Manual*, seventh edition [AJCC-7]) treated with nivolumab showed significant improvement in recurrence-free survival (RFS) compared with those treated with ipilimumab, an anti-cytotoxic T lymphocyte antigen-4 antibody (hazard ratio [HR] 0.68; 95% confidence interval [CI] 0.56–0.82; *P* < 0.0001; minimum follow-up, 36 months), with reduced toxicity [3]. In an updated analysis of CheckMate 238, the 5-year RFS and OS rates were 50% and 76%, respectively, among patients treated with nivolumab (minimum follow-up, 62.0 months) [4].

Data from real-world studies may complement results from RCTs by helping to address data gaps [5]. For example, comparing outcomes from RCTs with those from the real-world setting may provide important insights into the use of cancer treatments [6, 7]. Real-world evidence has been reported suggesting that adjuvant nivolumab treatment provides modest benefit in patients with resected stage IIIA melanoma [8–10]. The current comparative analysis aimed to validate clinical outcomes observed in patients with resected stage III melanoma who received adjuvant nivolumab in CheckMate 238 relative to a similar population from the real-world Flatiron Health electronic health record (EHR)-derived de-identified database. Time to treatment discontinuation and use of subsequent systemic treatment were also evaluated in the real-world cohort.

## Materials and methods

### Study design and data sources

This comparative analysis evaluated clinical outcomes in patients with completely resected stage III melanoma who received adjuvant nivolumab in either CheckMate 238 (NCT02388906; supplementary Data Sources) [2, 11] or in the real-world setting for up to 12 months, per label. Data for patients receiving 12 months of treatment and those receiving < 12 months were not analyzed separately. This analysis only included patients with completely resected stage III melanoma because patients with stage IV melanoma having no evidence of disease after resection are not included in the Flatiron Health database. Inclusion and exclusion criteria are shown in Table 1. Patients with a diagnosis of ocular/uveal melanoma prior to index date were excluded. The index date was the date of randomization to adjuvant nivolumab treatment for the CheckMate 238 cohort and the date of adjuvant nivolumab treatment initiation for the real-world cohort. In the CheckMate 238 cohort, patients who had resected stage III melanoma per AJCC-7 were reclassified per AJCC, eighth edition (AJCC-8). Data for the CheckMate 238 cohort were derived from the 5-year dataset (database lock, March 9, 2021). The real-world cohort was derived from the nationwide Flatiron Health EHR-derived de-identified database, which represents > 280 community cancer centers and eight major academic centers in the United States and includes more than three million records for patients being actively treated for cancer and followed longitudinally (supplementary Data Sources) [12]. Patients in the real-world cohort must have met the key eligibility criteria for the CheckMate 238 trial and were diagnosed with resectable stage III melanoma (per AJCC-8) between January 1, 2011, and June 30, 2022. The primary objectives of the study were to compare baseline characteristics between the two cohorts and to compare real-world OS (rwOS) in the Flatiron Health cohort with OS in the CheckMate 238 cohort.

**Table 1:** Inclusion and exclusion criteria for the CheckMate 238 and real-world cohorts

	CheckMate 238 cohort	Real-world cohort
*Inclusion criteria*		
Stage III melanoma diagnosis	Stage IIIA^a^ (only T1b/ulceration, N1–2a), stage IIIB (excluding T0), stage IIIC (excluding T0), or stage IIID melanoma based on AJCC-8	
Disease resection	Completely resected, histologically confirmed melanoma with no evidence of disease as per protocol	Initial diagnosis of resectable melanoma SLNB or CLND on or after the initial diagnosis Wide local excision of primary melanoma at the time of or in the 12 weeks prior to SLNB or lymphadenectomy
Adjuvant nivolumab administration^b^	Nivolumab 3 mg/kg Q2W Complete resection ≤ 12 weeks prior to randomization	Nivolumab 240 mg Q2W, 480 mg Q4W, or 3 mg/kg Q2W within the 12 weeks following the latest SLNB or lymphadenectomy (on or after December 20, 2017)
Other	Aged ≥ 15 years at index date^c^ ECOG PS 0–1	Aged ≥ 15 years at index date^c^ ECOG PS 0–1 or missing (assessed closest to and on or prior to index date)
*Exclusion criteria*		
Diagnosis of ocular/uveal melanoma prior to index date Nonmelanoma malignancies within 3 years prior to index date Autoimmune diseases prior to index date Conditions requiring systemic corticosteroids or other immunosuppressive medications within 14 days prior to index date Prior therapy for melanoma		

^a^Patients with AJCC-7 stage IIIA melanoma were not included in CheckMate 238; however, patients with T1b/ulceration, N1–2a, which is considered stage IIIA per AJCC-8, were allowed to enroll in the study

^b^Up to 12 months, per label (OPDIVO [package insert]. Princeton, *NJ* Bristol Myers Squibb Company; 2023)

^c^The index date was defined as the date of randomization to adjuvant nivolumab treatment in the CheckMate 238 cohort and the initiation date of the adjuvant nivolumab treatment in the real-world cohort

*AJCC-7* American Joint Committee on Cancer, *Cancer Staging Manual*, seventh edition, *AJCC-8* American Joint Committee on Cancer, *Cancer Staging Manual*, eighth edition, *CLND* complete lymph node dissection, *ECOG PS* Eastern Cooperative Oncology Group performance status, *N* node, *Q2W* every 2 weeks, *Q4W* every 4 weeks, *SLNB* sentinel lymph node biopsy, *T* tumor

### Outcomes

Baseline characteristics were assessed during screening (1–28 days before randomization) or at randomization in the CheckMate 238 cohort and during the 6-month period prior to the index date in the real-world cohort. Follow-up time in the CheckMate 238 cohort was defined as the period from the index date to death or date last known to be alive. Follow-up time in the real-world cohort was defined as the period from the index date to death or date of last confirmed activity (defined as the latest of the last confirmed structured activity or the last clinically relevant abstracted date [i.e., date of disease recurrence, metastasis, any oral therapy, specimen collection, medical procedure, clinical note, or disease progression]). OS in the CheckMate 238 cohort was defined as the time between the date of randomization and the date of death from any cause or the last date known to be alive. rwOS in the Flatiron Health cohort was defined as the time between the date of nivolumab initiation and the date of death from any cause; for patients without documentation of death, rwOS was censored at the data cutoff date (June 30, 2022). The mortality variable in the Flatiron Health database was curated from the following three sources: EHRs, the Social Security Death Index (SSDI), and obituary data. The mortality variable has been benchmarked to the recognized gold standard National Death Index across the 18 cancer types represented in Flatiron Health’s Enhanced Datamarts, which included advanced melanoma. The Flatiron Health mortality data have been determined to have high sensitivity (83.9%–91.5%), specificity (93.5%–99.7%), and positive predictive value (96.3%–98.3%) when benchmarked against SSDI data, all varying by tumor type [13]. Time to treatment discontinuation and use of subsequent systemic treatment were evaluated in the real-world cohort. Time to treatment discontinuation was defined as the time between the initiation of adjuvant nivolumab and treatment discontinuation for any reason (including death). Data for RFS and distant metastasis-free survival were not analyzed. Capturing or evaluating adverse events for nivolumab was outside of the scope of the analysis because safety data are not available in the Flatiron Health database.

### Statistical analysis

Baseline characteristics were compared between the two cohorts. Continuous variables for baseline characteristics were summarized using means and standard deviations (SDs) and compared using the Wald test. Categorical variables for baseline characteristics were summarized using frequency counts and percentages and compared using Chi-square tests (Fisher’s exact tests for variables with small frequency counts). Comorbidities with a prevalence rate of > 2% in the real-world cohort were evaluated.

OS in the CheckMate 238 cohort and rwOS in the Flatiron Health cohort were analyzed using the Kaplan–Meier method. rwOS was compared with OS using univariable (unadjusted) and multivariable (adjusted) Cox proportional hazards models, with calculation of hazard ratios (HRs) and associated 95% CIs. Median OS and rwOS, and their associated 95% CIs, were reported. Landmark OS and rwOS rates (e.g., at 1, 2, 3, and 4 years) were estimated. A multivariable Cox proportional hazards model was used to adjust for the following key prognostic factors: age, sex, race, disease stage, time from surgical resection to index date, Eastern Cooperative Oncology Group performance status (ECOG PS), and comorbidities of diabetes, chronic pulmonary disease, and atrial fibrillation (each with a prevalence of > 2% in the real-world cohort and known to be associated with increased mortality). Adjusted OS and rwOS Kaplan–Meier curves for the two cohorts were generated using the results of the Cox proportional hazards model, which was based on the Breslow method.

Inverse probability of treatment weighting (IPTW) [14] was used to reduce baseline discrepancies between the two cohorts and address residual confounding in the adjusted Cox proportional hazards model (supplementary IPTW Methods). IPTW aimed to achieve a balanced distribution of measured confounders at baseline across the cohorts, thereby simulating an RCT in which patients were randomly assigned to either study cohort. Weights were used to create a hypothetical sample in which the distribution of measured covariates was independent of the study cohorts. Weighting each patient created a “pseudo-population” in which the distribution of measured baseline covariates was similar between the two cohorts. Each patient was assigned a weight. Propensity scores were estimated using logistic regression as the probability of belonging to the CheckMate 238 cohort (vs. the real-world cohort) given an observed set of baseline covariates (i.e., age, sex, race [White or missing vs. non-White], disease stage [IIIC/D vs. IIIA/B], time from surgical resection to index date, ECOG PS [0 or missing vs. 1], diabetes, chronic pulmonary disease, and atrial fibrillation). Patients with missing race and/or ECOG PS were grouped into the most populated category of each specific variable (i.e., White race and ECOG PS 0).

Each patient’s weight was calculated as the inverse of the propensity score. Weights were stabilized using the marginal probability of being in their observed study cohort and truncated at the first and ninety-ninth percentiles. Stabilization of weights preserved the weighted total sample size so that it was similar to the original unweighted total sample size and increased the precision of estimates. A weighted multivariable Cox proportional hazards model was used to compare weighted rwOS with OS, adjusting for baseline characteristics. A standardized difference for a given baseline characteristic of < 0.1 was considered an inconsequential imbalance between the two cohorts [15]. If the standardized difference was > 0.1, that covariate was further adjusted for in the Cox model to address residual confounding.

Time to treatment discontinuation in the real-world cohort was analyzed using the Kaplan–Meier method. The number of patients in the real-world cohort initiating subsequent systemic treatment after the discontinuation of adjuvant nivolumab during the follow-up period was recorded.

All statistical analyses were conducted using SAS Enterprise Guide 7.1 software and R 3.6.3.

## Results

### Sample selection

**Fig. 1:**
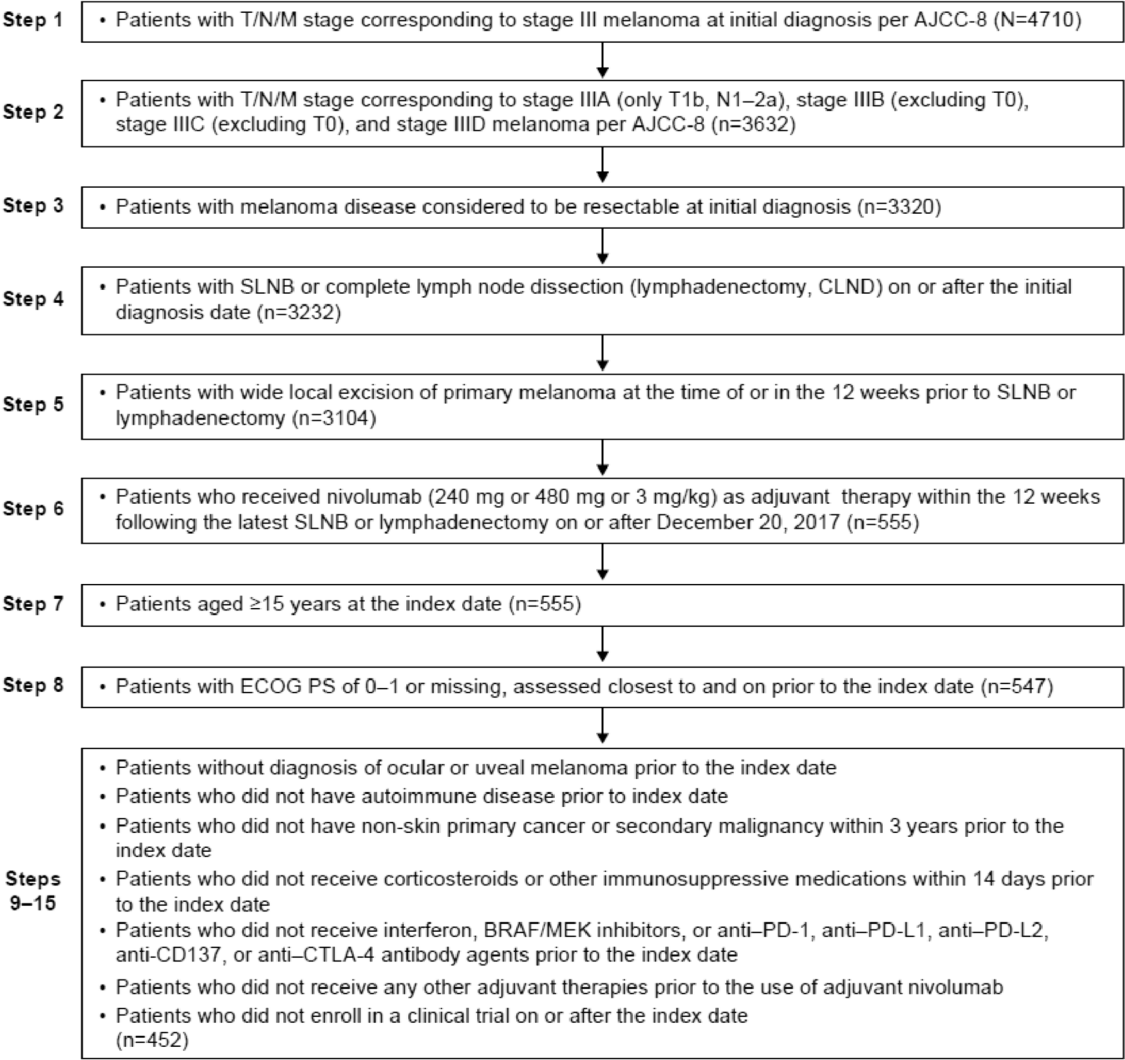
Sample selection in the real-world cohort

A total of 369 patients with resected stage III melanoma (per AJCC-8) receiving adjuvant nivolumab from the CheckMate 238 trial were included in the CheckMate 238 cohort. A total of 452 patients with resected stage III melanoma (per AJCC-8) who met key eligibility criteria for CheckMate 238 were included in the real-world cohort from the Flatiron Health database (Fig. 1).

### Baseline characteristics

The CheckMate 238 cohort, compared with the real-world cohort, had a lower median age (56.0 vs. 63.0 years; *P* < 0.001), lower median body weight (80.0 kg vs. 89.1 kg; *P* < 0.001), a lower proportion of patients with stage IIIA disease (1% [reclassified per AJCC-8] vs. 5%; *P* < 0.01 for differences in all disease stage categories), a longer mean time between surgical resection and index date (2.2 vs. 1.4 months; *P* < 0.001), and a lower proportion of patients with atrial fibrillation (1% vs. 4%; *P* < 0.05; Table 2). ECOG PS data were missing for no patient in the CheckMate 238 cohort and for 24% of patients in the real-world cohort. A higher percentage of patients were White in the CheckMate 238 cohort than in the real-world cohort (93% vs. 76%; *P* < 0.001 for all race categories). Patients in the CheckMate 238 cohort received nivolumab at 3 mg/kg every 2 weeks (Q2W), and patients in the real-world cohort received nivolumab at 3 mg/kg Q2W (1%), 240 mg Q2W (43%), or 480 mg every 4 weeks (56%; based on first dosing information or, if missing, the earliest available dosing information). *BRAF*-mutant disease was detected in 40% of patients in the CheckMate 238 cohort and 25% of patients in the real-world cohort, although *BRAF* mutation status data were missing in 17% and 36% of patients in the respective cohorts.

**Table 2:** Baseline characteristics in the CheckMate 238 and real-world cohorts

Characteristic	CheckMate 238 (*n* = 369)	Real-world (*n* = 452)	*P* value^a^
Age at index date, years, median (range)	56.0 (19–83)	63.0 (18.0–85.0)	< 0.001
Female – no. (%)	153 (41)	169 (37)	0.264
Race – no. (%)			
White	345 (93)	342 (76)	< 0.001
Asian	22 (6)	1 (< 1)	
Black or African American	0	2 (< 1)	
Other^b^	2 (1)	55 (12)	
Missing/no. (%)	0/369 (0)	52/452 (12)	
Body weight, kg, median (range)	80.0 (39.0–183.4)	89.1 (38.8–191.9)	< 0.001
Disease stage at initial diagnosis (per AJCC-8) – no. (%)			< 0.01
IIIA	3 (1)	21 (5)	
IIIB	117 (32)	136 (30)	
IIIC	232 (63)	276 (61)	
IIID	17 (5)	19 (4)	
Dosing – no. (%)			< 0.001
3 mg/kg Q2W	369 (100)	5 (1)	
240 mg Q2W	0	194 (43)	
480 mg Q4W	0	253 (56)	
Year of index date – no. (%)			< 0.001
2015	369 (100)	0	
2017	0	3 (1)	
2018	0	126 (28)	
2019	0	104 (23)	
2020	0	101 (22)	
2021	0	86 (19)	
2022	0	32 (7)	
Time from surgical resection to index date, months, mean ± SD	2.2 ± 0.6	1.4 ± 0.5	< 0.001
ECOG PS – no. (%)			< 0.001
0	333 (90)	271 (60)	
1	36 (10)	73 (16)	
Missing/no. (%)	0/369 (0)	108/452 (24)	
Comorbidities^c^ – no. (%)			
Diabetes	21 (6)	33 (7)	0.428
Chronic pulmonary disease	19 (5)	17 (4)	0.431
Atrial fibrillation	4 (1)	16 (4)	< 0.05
*BRAF* mutation status – no. (%)			< 0.05
Negative	157 (43)	178 (39)	
Positive	149 (40)	113 (25)	
Missing/no. (%)	63/369 (17)	161/452 (36)	

^a^Continuous variables were compared using the Wald test; categorical variables were compared using Chi-square tests (Fisher’s exact tests for variables with small frequency counts)

^b^Includes American Indian or Alaska Native, Hawaiian or Pacific Islander, or multiple races

^c^Those with a prevalence rate of > 2% in the real-world cohort

*AJCC-8* American Joint Committee on Cancer, *Cancer Staging Manual*, eighth edition, *ECOG PS* Eastern Cooperative Oncology Group performance status, *Q2W* every 2 weeks, *Q4W* every 4 weeks, *SD* Standard deviation

### Unadjusted OS and rwOS

**Fig. 2:**
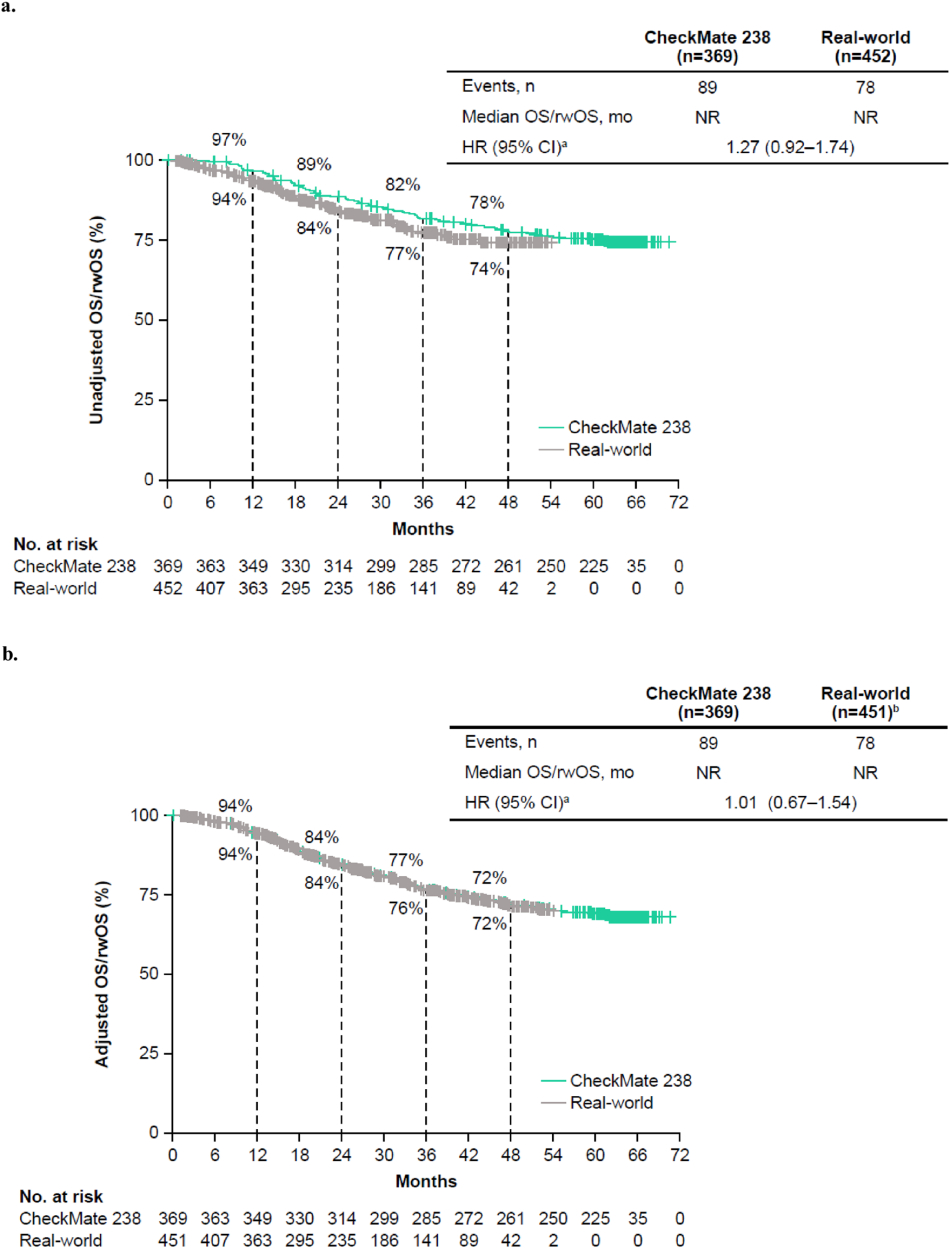
Unadjusted **a** and adjusted **b** OS in patients with resected stage III melanoma (per AJCC-8) who received adjuvant nivolumab in the CheckMate 238 and real-world cohorts, respectively. ^a^Comparison of real-world cohort versus CheckMate 238 cohort. ^b^451 of the 452 patients in the real-world cohort were included because one patient with missing comorbidity profiles was excluded *AJCC-8* American Joint Committee on Cancer, *Cancer Staging Manual*, eighth edition, *CI* Confidence interval, *HR* Hazard ratio, *NR* Not reached, *OS* Overall survival, *rwOS* Real-world overall survival

Median follow-up time (defined as the period from the index date to death or the last date known to be alive) was 61.4 months (range, 0.0–70.6) and 25.5 months (range, 0.8–54.1) in the CheckMate 238 and real-world cohorts, respectively. Deaths during the follow-up period occurred in 24% of patients (*n* = 89) in the CheckMate 238 cohort and 17% of patients (*n* = 78) in the real-world cohort. In the unadjusted analysis, rwOS was not different from OS (HR 1.27; 95% CI 0.92–1.74; Fig. 2a). OS rates were slightly higher than the rwOS rates across time points. Two-year OS and rwOS rates were 89% and 84%, respectively; 4-year OS and rwOS rates were 78% and 74%, respectively. Unadjusted median OS and rwOS were not reached in either cohort. In the unadjusted analysis, baseline covariates with significantly different rwOS compared with OS were age at the index date, sex (female vs. male), disease stage at initial diagnosis (IIIC/D vs. IIIA/B), ECOG PS (1 vs. 0), and diabetes (supplementary Table 1).

### Adjusted OS and rwOS using the Cox proportional hazards model

After adjusting for key prognostic factors (i.e., age, sex, race, disease stage, time from surgical resection to index date, ECOG PS, diabetes, chronic pulmonary disease, and atrial fibrillation) in the Cox proportional hazards model, rwOS was not different from OS (HR 1.01; 95% CI 0.67–1.54; Fig. 2b). Two-year OS rates were 84% in both cohorts; 4-year OS rates were 72% in both cohorts. Adjusted median rwOS and OS were not reached. Among the independent variables used in the Cox proportional hazards model, baseline covariates with significantly different (P < 0.05) rwOS compared with OS were age at index date and disease stage at initial diagnosis (IIIC/D vs. IIIA/B) (supplementary Table 2). Given that ECOG PS data were missing in 24% of patients in the real-world cohort, compared with 0% in the CheckMate 238 cohort, a sensitivity analysis was conducted that excluded patients with missing ECOG PS data, and the results from that analysis (HR 1.08; 95% CI 0.71–1.64) were consistent with those of the initial analysis (data not shown). For other variables included in the Cox model, missing data were rare.

### Adjusted OS and rwOS using the Cox proportional hazards model and IPTW

**Fig. 3:**
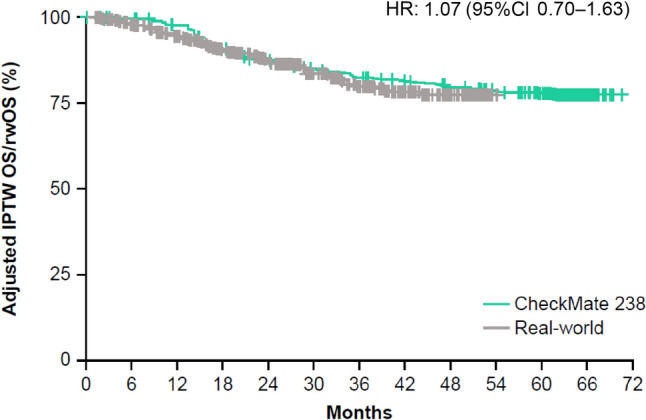
Adjusted IPTW OS and rwOS in patients with resected stage III melanoma (per AJCC-8) who received adjuvant nivolumab in the CheckMate 238 and real-world cohorts, respectively. *AJCC-8* American Joint Committee on Cancer, *Cancer Staging Manual*, eighth edition, *CI* Confidence interval, *HR* Hazard ratio, *IPTW* Inverse probability of treatment weighting, *OS* Overall survival, *rwOS* Real-world overall survival

A total of 820 patients, 369 from the CheckMate 238 cohort and 451 from the real-world cohort, were included in the logistic regression model for IPTW. (One patient with a missing comorbidity profile from the real-world cohort was excluded.) Baseline characteristics that were imbalanced between the two cohorts (with a standardized difference > 0.1) before IPTW were age at index date, race, time from surgical resection to index date, ECOG PS, and atrial fibrillation (Table 3). All the evaluated baseline characteristics were balanced between the two cohorts after IPTW, with the exception of time from surgical resection to index date, which was slightly longer in the CheckMate 238 cohort than in the real-world cohort (Table 3). After IPTW using stabilized truncated weights in a weighted Cox proportional hazards model, rwOS was not different from OS, with an adjusted HR after IPTW and after adjusting for time from surgical resection to the index date of 1.07 (95% CI 0.70–1.64; Fig. 3).

**Table 3:** Baseline characteristics before and after IPTW in the CheckMate 238 and real-world cohorts

	Before IPTW				After IPTW^a^			
	CheckMate 238 (*n* = 369)	Real-world (*n* = 451)^b^	Standardized difference^c^	*P* value	CheckMate 238 (*n* = 353.3)	Real-world (*n* = 416.5)	Standardized difference^c^	*P* value
*Patient characteristics*								
Age at index date, years, mean (SD)^d^	54.7 (13.4)	62.0 (14.0)	0.529	< 0.001	58.2 (13.5)	59.2 (15.1)	0.069	0.449
Sex								
Male	216 (58.5%)	282 (62.5%)	0.082	0.275	59.5%	59.5%	0.001	0.988
Female	153 (41.5%)	169 (37.5%)			40.5%	40.5%		
Race								
White or missing	345 (93.5%)	393 (87.1%)	0.216	< 0.01	90.6%	88.7%	0.064	0.512
Non-white	24 (6.5%)	58 (12.9%)			9.4%	11.3%		
*Disease characteristics*								
Disease stage at initial diagnosis (per AJCC-8)								
IIIA/IIIB	120 (32.5%)	156 (34.6%)	0.044	0.583	34.0%	32.6%	0.031	0.730
IIIC/IIID	249 (67.5%)	295 (65.4%)			66.0%	67.4%		
Time from surgical resection to index date, months, mean (SD)	2.2 (0.6)	1.4 (0.5)	1.410	< 0.001	1.8 (0.7)	1.7 (0.6)	0.263	< 0.01
ECOG PS								
0 or missing	333 (90.2%)	378 (83.8%)	0.192	< 0.01	87.0%	86.0%	0.028	0.768
1	36 (9.8%)	73 (16.2%)			13.0%	14.0%		
Comorbidities								
Diabetes	21 (5.7%)	33 (7.3%)	0.066	0.428	6.0%	6.5%	0.020	0.813
Chronic pulmonary disease	19 (5.1%)	17 (3.8%)	0.067	0.431	4.6%	4.0%	0.025	0.760
Atrial fibrillation	4 (1.1%)	16 (3.5%)	0.164	< 0.05	1.7%	2.6%	0.065	0.454

^a^The mean of stabilized truncated weights calculated from the propensity scores among patients in the CheckMate 238 and real-world cohorts was 0.96 (SD, 0.84) and 0.92 (SD, 0.62), respectively

^b^451 out of 452 patients in the real-world cohort were included because one patient with missing comorbidity profiles was excluded

^c^A standardized difference of < 0.1 was considered an inconsequential imbalance between the two cohorts

^d^The index date was defined as the date of randomization to adjuvant nivolumab treatment in the CheckMate 238 cohort and the initiation date of the adjuvant nivolumab treatment in the real-world cohort

*AJCC-8* American Joint Committee on Cancer, *Cancer Staging Manual*, eighth edition, *ECOG PS* Eastern Cooperative Oncology Group performance status, *IPTW* Inverse probability of treatment weighting, *SD* Standard deviation

### Time to treatment discontinuation and subsequent systemic therapy in the real-word cohort

Among the 452 patients in the real-world cohort, 340 (75%) discontinued treatment during the study period. The median time to treatment discontinuation in the real-world cohort was 10.4 months (95% CI 10.2–10.8), and the rate for remaining on treatment at 6 months was 72% (supplementary Fig. 1).

Among the 452 patients in the real-world cohort, 123 (27%) were reported to have received subsequent systemic therapy (supplementary Table 3). Among the 123 patients who received subsequent systemic therapy, 26 patients (21%) received subsequent treatment in the adjuvant setting, and 97 patients (79%) received subsequent treatment in the post-recurrence setting. The most common subsequent systemic therapies used in the real-world cohort were nivolumab plus ipilimumab (*n* = 34; 8%), nivolumab (*n* = 28; 6%), and dabrafenib plus trametinib (*n* = 17; 4%).

## Discussion

Results of this comparative analysis suggest that after adjustment, OS in the pivotal phase 3 CheckMate 238 trial [2] was similar to rwOS in the Flatiron Health database in patients with completely resected stage III melanoma (per AJCC-8) treated with adjuvant nivolumab, validating the results of the RCT. These findings are relevant given the limited real-world studies assessing the clinical outcomes of adjuvant treatments in patients with resected melanoma.

Baseline characteristics were generally similar between patients in the real-world Flatiron Health cohort (who met the key eligibility criteria for CheckMate 238) and those in the CheckMate 238 cohort, although there were a few notable differences. Compared with the CheckMate 238 cohort, the real-world cohort was older in age, possibly reflecting a lesser tendency to treat older patients with resected melanoma in the RCT (particularly because a high dose [10 mg/kg] of ipilimumab was used as the control treatment in CheckMate 238) and greater clinician experience with managing treatment-related toxicities in the real-world setting after regulatory approval of nivolumab. In addition, the real-world cohort had a slightly higher proportion of patients with stage IIIA disease per AJCC-8 than the CheckMate 238 cohort (5% vs. 1% [reclassified per AJCC-8]), which was due to selection criteria not allowing enrollment of patients with stage IIIA disease per AJCC-7 in CheckMate 238. Therefore, even when patients with low-risk, stage IIIB disease in CheckMate 238 were reclassified as having stage IIIA disease per AJCC-8, there were only a few patients with stage IIIA disease in the trial [16]. In addition, patients were more racially diverse in the real-world cohort than in the CheckMate 238 cohort, which may have reflected the underrepresentation of certain racial groups in the RCT. However, it is encouraging that results from a more racially diverse real-world cohort were consistent with RCT data.

The clinical benefit of adjuvant nivolumab observed in CheckMate 238 was similar to that observed in the real-world setting. Unadjusted and adjusted OS and rwOS in the CheckMate 238 and Flatiron Health cohorts, respectively, were not different, as 95% CIs for the HRs included 1. In the unadjusted analysis, the 2-year OS rate was similar to the 2-year rwOS rate (89% and 84%, respectively), as were 4-year OS and rwOS rates (78% and 74%, respectively), despite differences in baseline characteristics between the two populations. After applying similar patient selection criteria and adjusting for key prognostic factors, OS and rwOS rates remained similar between the cohorts (2-year OS and rwOS rates, 84% in both cohorts; 4-year OS and rwOS rates, 72% in both cohorts). In addition, OS and rwOS were not different after IPTW in the adjusted model, which controlled for residual differences between the two cohorts using a weighting approach. Furthermore, subsequent systemic therapy was used in similar percentages of patients in the nivolumab treatment arm in CheckMate 238 [2] and the real-world cohort (29% and 27%, respectively), suggesting that the use of subsequent systemic therapy did not influence the analysis. The results of this comparative analysis validate the OS benefit with adjuvant nivolumab observed in CheckMate 238 and suggest that those findings are generalizable beyond the RCT setting to the real-world setting.

This study had several limitations. As with any database analysis, there was the potential for errors in data entry and underreporting of clinical characteristics in the real-world database. Because disease conditions and comorbidities were defined by diagnosis codes in the real-word database, incompleteness or misclassification may have occurred. There was also the potential for incorrectly reported staging in the real-world cohort. Furthermore, there were complexities in extracting clinically relevant data for the real-world database using current EHR standards, which were largely designed for oncologists treating patients, tracking billing, and managing clinical care, even though strict quality assessment procedures served to maximize data integrity. The results may also have been influenced by unobserved prognostic factors that were not accounted for in the multivariable analysis, such as sentinel lymph node tumor burden in patients with IIIA disease, as this information was not captured in CheckMate 238. Moreover, the limited follow-up in patients with a relatively good prognosis was likely to have resulted in substantial censoring of survival outcomes due to improved outcomes in the real-world setting. The efficacy analysis may have been affected by differences in the definitions for OS in the CheckMate 238 cohort (time between randomization [index date] and death or date last known to be alive) and rwOS in the real-world cohort (time between nivolumab initiation [index date] and death or data cutoff). Given that the real-world database did not have information describing reasons for censoring, rwOS was censored at the data cutoff date. However, this methodology may have potentially overestimated the time at risk close to data cutoff. The findings of this analysis may have also been affected by missing data in the real-world cohort. For example, ECOG PS data were missing in 24% of patients in the real-world cohort, whereas none of the patients in the CheckMate 238 cohort had missing ECOG PS data. However, the results from a sensitivity analysis that excluded patients with missing ECOG PS data were consistent with those of the initial analysis. Median follow-up time also differed substantially between the CheckMate 238 and the real-world cohorts (61.4 vs. 25.5 months). Although patients were monitored regularly for outcome assessment in CheckMate 238, it is unclear how frequently patients were monitored in the real-world setting, which is an important factor in observing recurrences. Finally, this analysis may have been affected by geographic limitations of the flow of data into the Flatiron Health database. Despite these limitations, this analysis provides insights into clinical outcomes with adjuvant nivolumab in patients with resected melanoma in routine clinical practice.

In this comparative analysis involving patients with completely resected stage III melanoma (per AJCC-8) treated with adjuvant nivolumab, OS in the phase 3 CheckMate 238 trial was similar to rwOS in the Flatiron Health database, validating results from the RCT. These findings suggest that results from CheckMate 238 are generalizable to the real-world setting and support adjuvant nivolumab as a standard of care for this patient population.

### Supplementary Information

Below is the link to the electronic supplementary material.

Supplementary file1 (DOCX 211 KB)

## Data Availability

The Bristol Myers Squibb policy on data sharing may be found at https://www.bms.com/researchers-and-partners/clinical-trials-and-research/disclosure-commitment.html.

## Abbreviations

AJCC: American Joint Committee on Cancer
CI: Confidence interval
ECOG PS: Eastern Cooperative Oncology Group performance status
EHR: Electronic health record
HR: Hazard ratio
I-O: Immuno-oncology
IPTW: Inverse probability of treatment weighting
OS: Overall survival
PD-1: Programmed cell death-1
RCT: Randomized controlled trial
RFS: Recurrence-free survival
rwOS: Real-world overall survival
