# Supplementary material for: Clinical outcomes of adjuvant nivolumab in resected stage III melanoma: comparison of CheckMate 238 trial and real-world data
Source: Cancer Immunol Immunother. 2024 May 7;73(7):116. doi: 10.1007/s00262-024-03697-3 (PMC11076438; doi:10.1007/s00262-024-03697-3)

**Supplement**

Cancer Immunology, Immunotherapy (submitted in 2023) - Justin C. Moser et al.

**Data sources**

The CheckMate 238 cohort was derived from the CheckMate 238 trial (NCT02388906), a randomized, double-blind, phase 3 study of adjuvant therapy with nivolumab versus ipilimumab in patients with completely resected stage IIIB/C or stage IV melanoma per American Joint Committee on Cancer, *Cancer Staging Manual*, seventh edition (AJCC-7) [1, 2]. A total of 906 patients aged 15 years or older were randomly assigned from March 30, 2015, to November 30, 2015, in a 1:1 ratio to receive either nivolumab 3 mg/kg intravenously every 2 weeks (n=453) or ipilimumab 10 mg/kg intravenously every 3 weeks for 4 doses followed by every 12 weeks (n=453). Patients were stratified by programmed death ligand 1 status and stage according to AJCC-7, Patients were treated for up to 1 year or until disease recurrence, unacceptable toxicity, or consent to withdraw. Recurrence-free survival was the primary endpoint, overall survival was a key secondary endpoint, and distant metastasis-free survival was an exploratory endpoint. Patients in the CheckMate 238 cohort in this comparative analysis had completely resected stage III melanoma reclassified per American Joint Committee on Cancer, *Cancer Staging Manual*, eighth edition (AJCC-8). Patients with stage IV melanoma were excluded in CheckMate 238 given that their disease was unresectable. Data for the CheckMate 238 cohort were derived from the 5-year dataset (database lock, March 9, 2021).

The real-world cohort was derived from the nationwide Flatiron Health electronic health record (EHR)-derived de-identified database, which represents >280 community cancer centers and eight major academic centers in the United States and includes more than three million records for patients being actively treated for cancer and followed longitudinally [3]. Data in the Flatiron Health database are updated monthly and contain data elements extracted from structured EHRs that are mapped to a common terminology and normalized across different source systems (ie, normalized data) and unstructured information abstracted from physicians’ notes and other unstructured documents such as radiology, pathology, and biomarker reports, and patient discharge summaries (ie, enhanced and derived data). Structured and unstructured data included in the Flatiron Health database can be traced back to the source EHR to ensure accuracy of data. The normalized data elements include demographics, diagnoses, visits, laboratory and vital sign data, medication administration, medication orders/prescriptions, performance status, and insurance data. The advanced melanoma-specific module contains additional disease-specific data abstracted from patient charts, including dates of locoregional recurrence and distant recurrence, and biomarker test results. Additionally, the Flatiron Health database provides validated mortality data that are curated from both structured and unstructured EHR fields, as well as external sources such as the Social Security Death Index (SSDI), and a commercial death dataset that derives data from obituaries, funeral homes, and other sources to provide death data that is current within a week of the death. The Flatiron Health mortality data have been determined to have high sensitivity (83.9%–91.5%), specificity (93.5%–99.7%), and positive predictive value (96.3%–98.3%) when benchmarked against SSDI data, all varying by tumor type [4]. Patients in the real-world cohort in this study met key eligibility criteria for the CheckMate 238 trial, had resected stage III melanoma per AJCC-8, and were diagnosed with advanced melanoma between January 1, 2011, and June 30, 2022.

**Inverse Probability of Treatment Weighting (IPTW) methods**

IPTW [5] was used to reduce baseline discrepancies and address residual confounding in the adjusted Cox proportional hazards model between the two cohorts. Conceptually, the IPTW approach aimed to achieve a balanced distribution of measured confounders across study cohorts, thereby simulating a randomized controlled trial whereby patients were randomly assigned to either cohort. IPTW used weights to create a hypothetical sample in which the distribution of measured covariates was independent of study cohorts. Using IPTW to weight each patient created a “pseudo-population” in which the distribution of measured baseline covariates was similar between study cohorts (**Figure**).

**Figure** Applying IPTW to achieve balance in study cohorts.

**
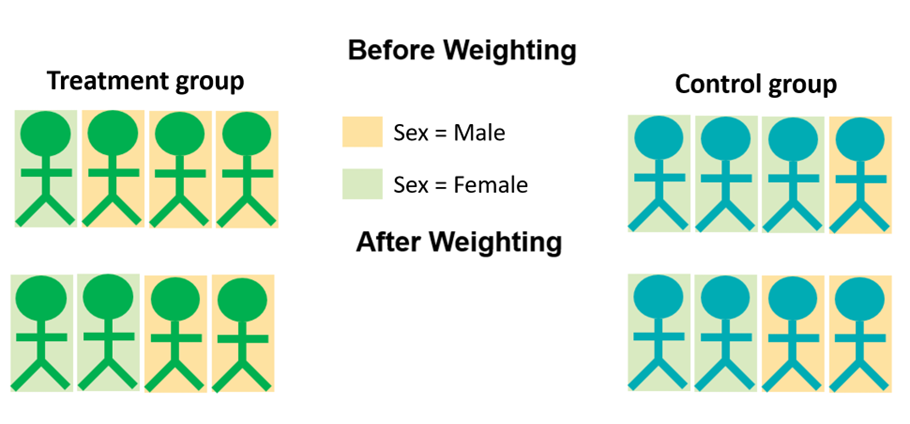
**

To implement IPTW, weights were estimated from propensity score models, in which the propensity score was defined as the probability of being in a specific study cohort given an observed set of baseline covariates. Each observation’s weight was calculated as the inverse of the conditional probability of being in their observed study cohort (ie, CheckMate 238 cohort or real-world cohort) given their covariates. It was estimated using a multivariable logistic regression model in which being in the CheckMate 238 cohort was the dependent variable (and being in the real-world cohort served as the reference) and a set of measured baseline covariates (ie, age, sex, race, disease stage, time from surgical resection to index date, Eastern Cooperative Oncology Group performance status [ECOG PS], diabetes, chronic pulmonary disease, and atrial fibrillation) were included as independent variables. Patients in the CheckMate 238 cohort were weighted by $1/{Pr(C=1 | V = v)}$and patients in the real-world cohort were weighted by $1/{(1-Pr[C=1 | V = v])}$, in which C was the study cohort (1 represented the CheckMate 238 cohort), and V was a vector of covariates. By weighting the model as such, a “pseudo-population” was created in which a patient’s study cohort was independent of measured baseline covariates.

To enhance precision in point estimates and adjust influence from patients with extreme weights, each observation’s weight was stabilized by the marginal probability of being in their study cohort. The stabilized weights were thus calculated as follows:

$$\left( 1 \right) \frac{Pr(C=1)}{\Pr\left( C=1 \right|V = v)} for the CheckMate 238 cohort$$

$$\left( 2 \right) \frac{1- Pr(C=1)}{1- \Pr\left( C=1 \right|V = v)} for the real world cohort$$

The distribution of the stabilized weights was evaluated for extreme values by assessing the mean, standard deviation, and minimum and maximum values. The weights were truncated at the first and ninety-ninth percentiles to minimize the impact of extreme values. After IPTWs were calculated, the distribution of baseline characteristics was compared between the weighted study cohorts using standardized differences to ensure that they were balanced. For continuous variables, the standardized difference was calculated using the following equation in which $\bar{x}$ was the respective mean and $s^{2}$ was the respective variance of the variable in each study cohort:

$$\frac{|\bar{x}_{CheckMate 238}-\bar{x}_{real world}|}{\sqrt{\frac{1}{2}({s^{2}}_{CheckMate 238}+ {s^{2}}_{real world})}}$$

For dichotomous variables, the standardized difference was calculated using the following equation in which P was the respective proportion of participants in each study cohort:

$$\frac{|\hat{P}_{CheckMate 238}-\hat{P}_{real world}|}{\sqrt{{\frac{1}{2}(\hat{P}}_{CheckMate 238}\times\left( 1-\hat{P}_{CheckMate} \right)+\hat{P}_{real wold}\times(1-\hat{P}_{real world}))}}$$

Next, a Cox proportional hazards model with time to death as the dependent variable and being in the CheckMate 238 cohort as the independent variable was fitted using the weighted study population to compare weighted real-world overall survival with weighted overall survival in the CheckMate 238 cohort. If the standardized difference was >0.1 for a given baseline characteristic, that covariate was further adjusted for in the Cox model to address residual confounding. A standardized difference of <0.1 is generally recognized as an inconsequential imbalance between the two populations.[6]

**References**

1. Weber J, Mandala M, Del Vecchio M, Gogas HJ, Arance AM, Cowey CL, et al, for the CheckMate 238 Collaborators (2017) Adjuvant nivolumab versus ipilimumab in resected stage III or IV melanoma. N Engl J Med 377:1824–1835. https://doi.org/10.1056/NEJMoa1709030
2. Ascierto PA, Del Vecchio M, Mandalá M, Gogas H, Arance AM, Dalle S, Cowey CL, Schenker M, Grob J-J, Chiarion-Sileni V, Márquez-Rodas I, Butler MO, Maio M, Middleton MR, de la Cruz-Merino L, Arenberger P, Atkinson V, Hill A, Fecher LA, Millward M, Khushalani NI, Queirolo P, Lobo M, de Pril V, Loffredo J, Larkin J, Weber J (2020) Adjuvant nivolumab versus ipilimumab in resected stage IIIB-C and stage IV melanoma (CheckMate 238): 4-year results from a multicentre, double-blind, randomised, controlled, phase 3 trial. Lancet Oncol21:1465–1477. https://doi.org/10.1016/S1470-2045(20)30494-0
3. Flatiron. Quick Facts. Available: https://flatiron.com/media [Accessed 26 Jun 2023]
4. Zhang Q, Gossai A, Monroe S, Nussbaum NC, Parrinello CM (2021) Validation analysis of a composite real-world mortality endpoint for patients with cancer in the United States. Health Serv Res 56:1281–1287. https://doi.org/10.1111/1475-6773.13669
5. Xu S, Ross C, Raebel MA, Shetterly S, Blanchette C, Smith D (2010) Use of stabilized inverse propensity scores as weights to directly estimate relative risk and its confidence intervals. Value Health 13:273–277. https://doi.org/10.1111/j.1524–4733.2009.00671.x
6. Austin PC (2009) Using the standardized difference to compare the prevalence of a binary variable between two groups in observational research. Commun Stat Simul Comput 38:1228–1234. https://doi.org/10.1080/03610910902859574

**Supplementary Table 1** HRs for the independent variables in the unadjusted Cox proportional hazards model

| **Effect** | **HR (95% CI)** | **P value** |
| --- | --- | --- |
| Data source (real-world cohort vs. CheckMate 238 cohort) | 1.27 (0.92–1.74) | 0.14 |
| Age at the index date (years) | 1.02 (1.01–1.03) | <0.001 |
| Sex (female vs. male) | 0.70 (0.51–0.98) | <0.05 |
| Race categorization (non-White vs. White) | 1.31 (0.78–2.20) | 0.31 |
| Race categorization (missing vs. White) | 1.72 (0.93–3.18) | 0.09 |
| Disease stage at initial diagnosis (IIIC/D vs. IIIA/B; per AJCC-8) | 1.97 (1.36–2.85) | <0.001 |
| Time from surgical resection to index date^a^ (months) | 0.89 (0.72–1.10) | 0.28 |
| ECOG PS (1 vs. 0) | 1.68 (1.12–2.52) | <0.05 |
| ECOG PS (missing vs. 0) | 1.10 (0.67–1.82) | 0.70 |
| Diabetes | 1.68 (1.02–2.77) | <0.05 |
| Chronic pulmonary disease | 0.77 (0.34–1.74) | 0.53 |
| Atrial fibrillation | 2.10 (0.93–4.76) | 0.07 |

^a^The index date was defined as the date of randomization to adjuvant nivolumab treatment in the CheckMate 238 cohort and the initiation date of the adjuvant nivolumab treatment in the real-world cohort

*AJCC-8* American Joint Committee on Cancer, *Cancer Staging Manual*, eighth edition,
*CI* confidence interval, *ECOG PS* Eastern Cooperative Oncology Group performance status, *HR* hazard ratio, *OS* overall survival, *rwOS* real-world overall survival

**Supplementary Table 2** HRs for the independent variables in the adjusted Cox proportional hazards model

| **Effect** | **HR (95% CI)** | **P value** |
| --- | --- | --- |
| Data source (real-world cohort vs. CheckMate 238 cohort) | 1.01 (0.67–1.54) | 0.95 |
| Age at the index date (years) | 1.02 (1.00–1.03) | <0.05 |
| Sex (female vs. male) | 0.79 (0.57–1.10) | 0.16 |
| Race categorization (non-White vs. White) | 1.23 (0.73–2.08) | 0.44 |
| Race categorization (missing vs. White) | 1.57 (0.82–3.00) | 0.17 |
| Disease stage at initial diagnosis (IIIC/D vs. IIIA/B; per AJCC-8) | 1.84 (1.27–2.67) | <0.01 |
| Time from surgical resection to index date^a^ (months) | 0.99 (0.76–1.28) | 0.92 |
| ECOG PS (1 vs. 0) | 1.39 (0.92–2.11) | 0.12 |
| ECOG PS (missing vs. 0) | 0.97 (0.56–1.67) | 0.91 |
| Diabetes | 1.37 (0.82–2.29) | 0.22 |
| Chronic pulmonary disease | 0.69 (0.30–1.59) | 0.39 |
| Atrial fibrillation | 1.69 (0.72–3.93) | 0.23 |

^a^The index date was defined as the date of randomization to adjuvant nivolumab treatment in the CheckMate 238 cohort and the initiation date of the adjuvant nivolumab treatment in the real-world cohort

*AJCC-8* American Joint Committee on Cancer, *Cancer Staging Manual*, eighth edition,
*CI* confidence interval, *ECOG PS* Eastern Cooperative Oncology Group performance status, *HR* hazard ratio, *OS* overall survival, *rwOS* real-world overall survival

**Supplementary Table 3** Subsequent systemic treatment in the real-world cohort

|  | **All patients (n=452)** | ***BRAF* negative (n=178)** | ***BRAF* positive (n=113)** |
| --- | --- | --- | --- |
| Subsequent treatment – no. (%) |  |  |  |
| Did not receive subsequent treatment | 329 (73) | 134 (75) | 73 (65) |
| Received subsequent treatment | 123 (27) | 44 (25) | 40 (35) |
| Subsequent treatment distribution – no. (%) |  |  |  |
| Ipilimumab, nivolumab | 34 (8) | 15 (8) | 3 (3) |
| Nivolumab | 28 (6) | 14 (8) | 5 (4) |
| Dabrafenib, trametinib | 17 (4) | 0 | 15 (13) |
| Binimetinib, encorafenib | 10 (2) | 2 (1) | 8 (7) |
| Ipilimumab | 7 (2) | 6 (3) | 0 |
| Pembrolizumab | 7 (2) | 3 (2) | 2 (2) |
| Ipilimumab, pembrolizumab | 2 (<1) | 0 | 1 (1) |
| Talimogene laherparepvec | 2 (<1) | 1 (1) | 1 (1) |
| Abiraterone | 1 (<1) | 0 | 0 |
| Binimetinib, encorafenib, ipilimumab, nivolumab | 1 (<1) | 0 | 1 (1) |
| Capecitabine | 1 (<1) | 0 | 0 |
| Cetuximab, dabrafenib, trametinib | 1 (<1) | 0 | 0 |
| Cobimetinib, vemurafenib | 1 (<1) | 0 | 1 (1) |
| Cyclophosphamide, doxorubicin | 1 (<1) | 1 (1) | 0 |
| Fluorouracil | 1 (<1) | 0 | 0 |
| Fluorouracil, nivolumab | 1 (<1) | 0 | 1 (1) |
| Ibrutinib, nivolumab | 1 (<1) | 0 | 0 |
| Ibrutinib, venetoclax | 1 (<1) | 1 (1) | 0 |
| Letrozole | 1 (<1) | 0 | 1 (1) |
| Letrozole, nivolumab | 1 (<1) | 0 | 0 |
| Nivolumab, talimogene laherparepvec | 1 (<1) | 1 (1) | 0 |
| Obinutuzumab, venetoclax | 1 (<1) | 0 | 1 (1) |
| Paclitaxel | 1 (<1) | 0 | 0 |
| Venetoclax | 1 (<1) | 0 | 0 |

**Supplementary Figure 1** Kaplan–Meier analysis for time to treatment discontinuation in the real-word cohort.


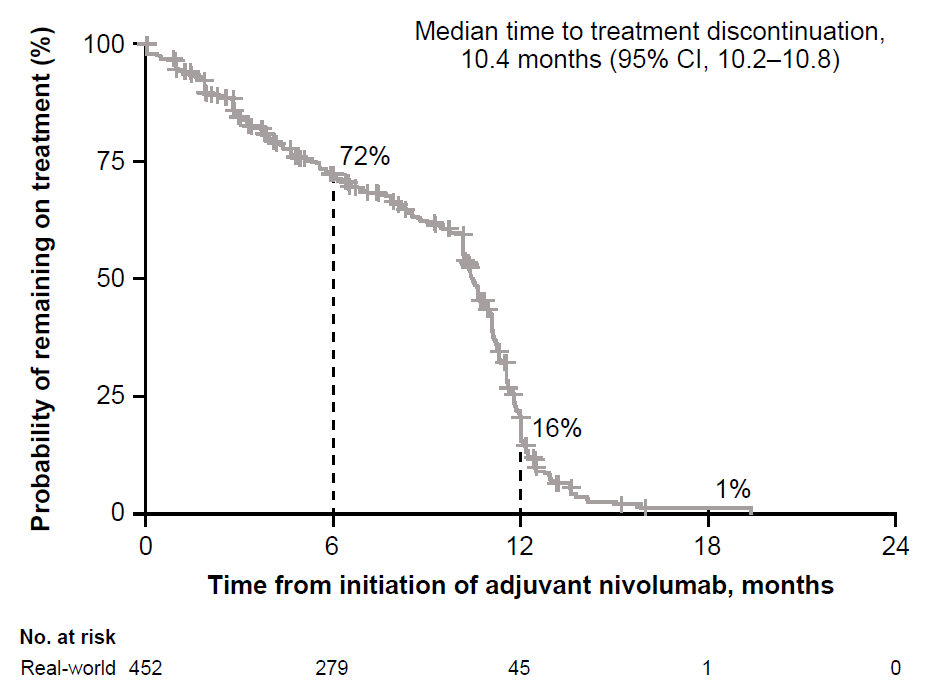

Supplement: Supplementary file 1 — Supplementary file1 (DOCX 211 KB) [file 262_2024_3697_MOESM1_ESM.docx]
